# Cervical metastasis of breast cancer: A case report and literature review

**DOI:** 10.1097/MD.0000000000039275

**Published:** 2024-08-09

**Authors:** Lu Zhang, Xi Yang, Yuanyuan Zhang, Yaru Wen, Manni Huang, Jusheng An

**Affiliations:** a Department of Gynecological Oncology, National Cancer Center/National Clinical Research Center for Cancer/Cancer Hospital, Chinese Academy of Medical Sciences and Peking Union Medical College, Beijing, China; b Department of Pathology, National Cancer Center/National Clinical Research Center for Cancer/Cancer Hospital, Chinese Academy of Medical Sciences and Peking Union Medical College, Beijing, China.

**Keywords:** breast cancer, HDR brachytherapy, metastatic cervical cancer

## Abstract

**Rationale:**

Cervical metastasis of breast cancer is rare and its clinical manifestations are similar to those of primary cervical cancer. It is thus easy to misdiagnose, with diagnosis mainly depending on pathology and immunohistochemistry. There have been few studies on its treatment and there is thus no standard treatment plan.

**Patient concerns:**

This is a 64-year-old female patient presented with a 2-month history of abnormal postmenopausal vaginal discharge, who had previous history of breast cancer.

**Diagnoses:**

Based on the gynecological examination, imaging results, pathology, and immunohistochemical results, a diagnosis of metastatic carcinoma of the cervix and breast cancer was confirmed.

**Interventions:**

She received computed tomography-guided 3-dimensional high-dose-rate brachytherapy in combination with chemotherapy.

**Outcomes:**

She achieved complete response locally. This case provides a new local treatment option for patients with inoperable localized cervical metastases.

**Lessons:**

We hope that this report and the accompanying review help to enrich the literature pertaining to the treatment of rare cervical metastases, providing a foundation for the improved survival of affected patients.

## 1. Introduction

Cervical cancer is a common gynecological malignancy, with metastatic cervical tumors most commonly originating from female reproductive tumors including endometrial or ovarian cancer. Chen and Wang^[1]^ also reported that cancers of the gastrointestinal tract also commonly metastasize to the cervix, while Pérez-Montiel et al^[2]^ reported metastases being common for ovarian and gastrointestinal tumors. Cervical metastasis occurs in breast cancer patients with an incidence rate of 0.8% to 1.7%.^[3,4]^ In this report, we describe a case of breast cancer metastasis to the cervix. This case is presented along with a discussion of the recent literature pertaining to the diagnosis, treatment, and prognosis of breast cancer metastasis to the cervix with the goal of improving current knowledge of this form of metastatic disease.

## 2. Case report

### 2.1. Patient information

**Figure 1.**
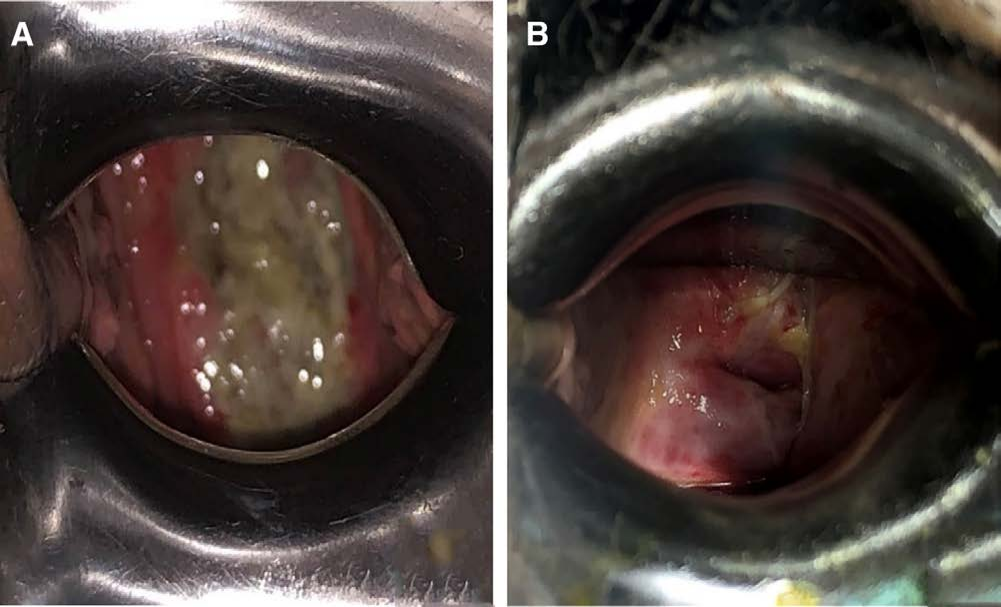
(A) The gross observation of local cervical tumor before HDR-BT. (B) The gross observation of local cervical tumor before HDR-BT. HDR-BT = high-dose-rate brachytherapy.

**Figure 2.**
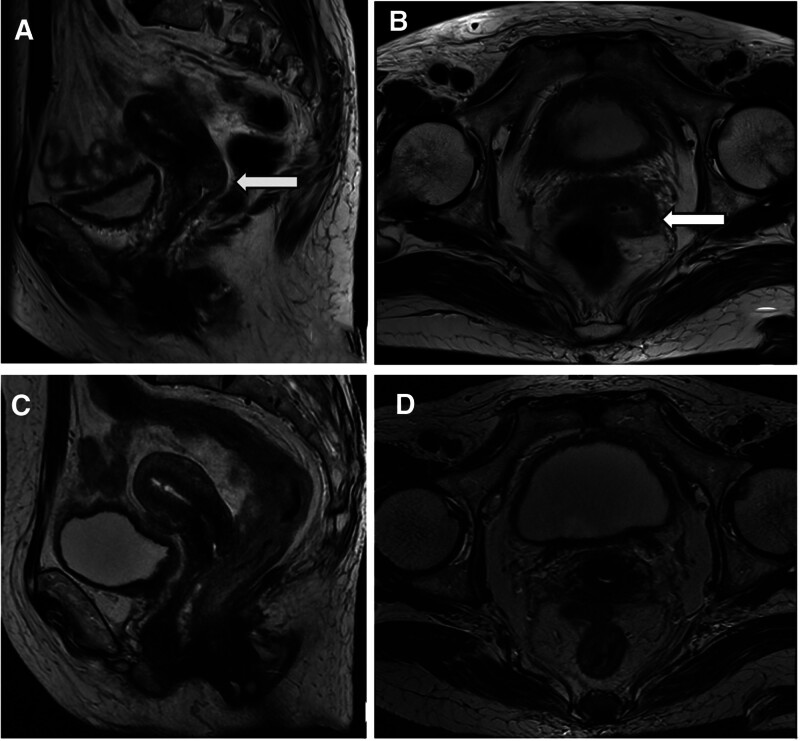
(A) Sagittal MR view of pelvis before HDR-BT. (B) Coronal MR view of pelvis before HDR-BT. (C) Sagittal MR view of pelvis after HDR-BT. (D) Coronal MR view of pelvis after HDR-BT. HDR-BT = high-dose-rate brachytherapy, MR = magnetic resonance.

A 64-year-old female patient presented with a 2-month history of abnormal postmenopausal vaginal discharge. The patient reportedly began experiencing an increase in vaginal secretions in August 2022 without inducement, with these secretions being of substantial volume, yellow, thin, accompanied by an odor, but not accompanied by vaginal bleeding. On August 13, 2022, the patient presented to a local hospital where she was found to be human papillomaviruses negative and thinprep cytologic test normal, with cervical biopsy pathology results yielding a diagnosis of poorly differentiated squamous cell carcinoma. When the patient visited our hospital on September 6, 2022, gynecological examination was performed with the following results (Fig. 1A): vulva: (−); the vagina was unobstructed, with the left side and posterior fornix exhibiting invasive contractures; the cervix was 4.5 cm in diameter, with a nodular anterior lip and a posterior lip that appeared cauliflower-like, an external orifice that was ulcerated and cavitated, bleeding on palpation (+), 2.0 cm protrusion, cervical canal thickness of 4.0 cm, and medium quality; the uterus was in a middle anterior position with enlargement; the left side of the uterus was slightly shortened with thick cord-like structures and medium elasticity; while the right side of the uterus exhibited sheet-like thick morphology and elasticity; and light rectal mucosa. Pathology results following the biopsy of the identified cervical mass revealed a poorly differentiated carcinoma. Magnetic resonance imaging supported a diagnosis of cervical cancer with suspected vaginal fornix involvement (Fig. 2A, B), and concern was raised regarding the potential for metastasis to the left paracervical and left iliac paracervical lymph nodes.

#### 2.1.1. History

On March 13, 2018, the patient had undergone modified radical mastectomy of the right breast in another hospital due to a diagnosis of cancer of the right breast. The postoperative pathology results from that procedure supported a diagnosis of breast ductal carcinoma in situ, with 0/24 positive axillary lymph nodes. Immunohistochemistry (IHC) results for the primary tumor were as follows: C-erbB-2 (intraductal 1 +), ER (+), PR (+). The patient underwent postoperative radiotherapy, although the details of the treatment regimen are unknown, and she was treated with herceptin (580 mg) for 1 year beginning on March 27, 2018 to March 2019. She began to take anastrozole orally at some time in 2019 (dose unknown) but she discontinued its use in November 2020. On November 6, 2020, the patient underwent modified radical mastectomy for cancer of the left breast, with postoperative pathology yielding a diagnosis of invasive breast cancer. Intravascular tumor emboli and metastases were detected in analyzed lymph nodes (35/35). IHC results were as follows: C-erbB-2 (3 +), ER (−, <1% weak positive), PR (−, < 1% weak positive). The patient was administered 19 cycles of chemotherapy (paclitaxel + pertuzumab + trastuzumab) from December 11, 2020 to December 26, 2021. Owing to issues with pertuzumab reimbursement, the patient then underwent 7 cycles of maintenance chemotherapy with the TH (paclitaxel + pertuzumab) regimen from January 16, 2022 to June 18, 2022. Owing to the extended duration of maintenance treatment, the patient experienced pronounced side effects such that only trastuzumab single-target therapy was continued for 5 cycles from July 8, 2022 to September 23, 2022.

### 2.2. Diagnostic assessment

**Figure 3.**
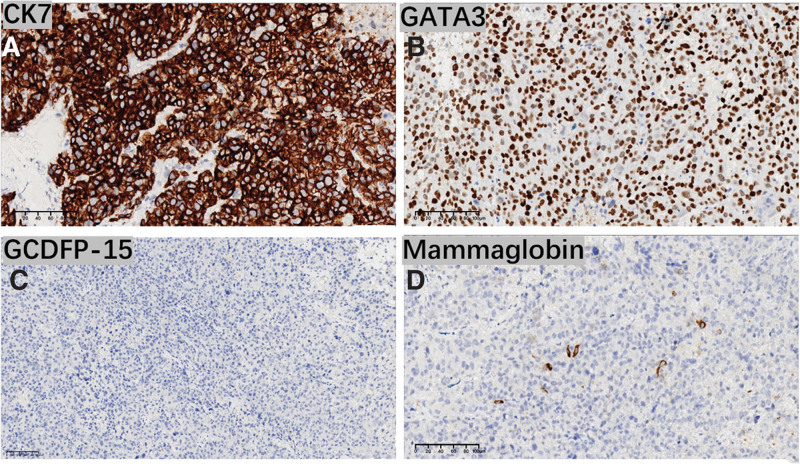
Immunohistochemistry for cervical and breast cancer-related pathology. (A) Cytokeratin 7 (B) Gata binding protein 3 (C) Grosscystic disease fluid protein-15 (D) Mammaglobin.

Based on the gynecological examination and imaging results, the patient was diagnosed with stage IIIC1r cervical cancer and was admitted to the hospital for concurrent radiotherapy and chemotherapy. Based on the pathology and immunohistochemical results from the cervical tissue together with the patient’s medical history, this case was considered to be consistent with metastatic breast cancer. Cervical IHC results were as follows: CK 7 (3 +), GATA3 (3 +), mammaglobin (few positive), GCDFP15 (−), ER (−), PR (30% weakly positive). P16 (−), P40 (−), P63 (−), PAX8 (−) (Fig. 3). Based on these results, a revised diagnosis of metastatic carcinoma of the cervix and breast cancer was confirmed. Positron emission tomography-computed tomography imaging revealed multiple systemic metastases, involving metastases present in the esophagus and mediastinal lymph nodes.

### 2.3. Therapeutic intervention

**Figure 4.**
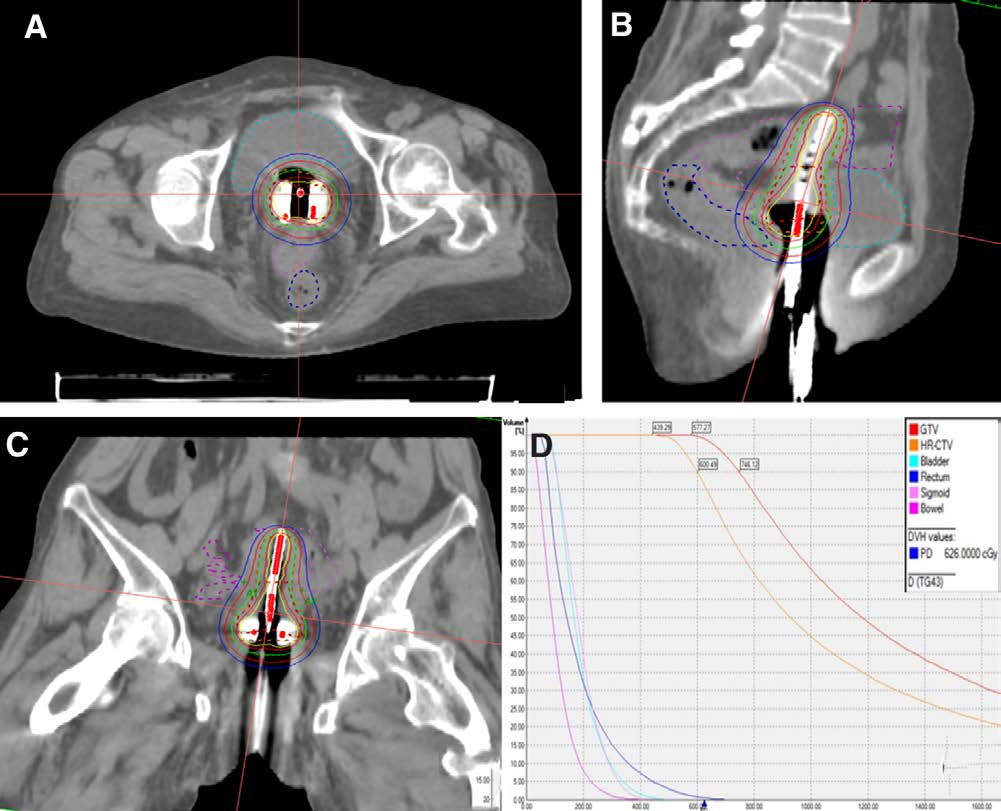
Three-dimensional HDR-BT dose line and DVH diagram. (A) Transverse bit image (B) Sagittal images (C) Coronal image (D) DVH diagram. DVH = dose and volume histogram, HDR-BT = high-dose-rate brachytherapy.

Multidisciplinary team consultation and discussion led to the determination that the local cervical tumors had likely progressed rapidly, invading the uterus and disseminating throughout the body. The potential for complete surgical resection was regarded as being very small and the procedure was considered difficult such that palliative cervical radiotherapy was recommended. From October 19, 2022 to November 17, 2022, 6 rounds of image-guided 3-dimensional brachytherapy were performed. Cumulative dose (equivalent dose in 2Gy/f): HR-CTV D90 48.0 Gy, A1 23.4Gy, A2 25.8Gy. Normal tissue limits: rectum: D2cc 33.0 Gy, sigmoid: D2cc 27.6 Gy, bladder: D2cc 33.6 Gy (see Fig. 4 and Table 1 for target volume and dose).

**Table 1:** Brachytherapy dose values.

ROI	Dose (%)	Dose (cGy)	Volume (%) (&)	Volume (ccm)
Bladder	80.71	484.26	0.09	0.1
Bladder	71.34	428.03	0.86	1.0
Bladder	66.37	398.23	1.72	2.0
Bowel	68.45	410.72	0.06	0.1
Bowel	54.78	328.67	0.56	1.0
Bowel	50.01	300.05	1.13	2.0
GTV	95.68	574.06	100	26.99
GTV	124.25	745.50	90	24.29
GTV	107.68	646.10	98	26.45
HR-CTV	73.87	443.23	100	61.07
HR-CTV	100.04	600.23	90	54.96
HR-CTV	87.81	526.84	98	54.96
Rectum	107.64	645.81	0.38	0.1
Rectum	79.64	477.24	3.8	1.0
Rectum	65.77	394.64	7.6	2.0
Sigmoid	74.04	444.25	0.12	0.1
Sigmoid	62.82	376.90	1.16	1.0
Sigmoid	58.97	353.80	2.32	2.0

GTV = gross target volume, HR-CTV = high risk clinical target volume, ROI = regions of interest.

### 2.4. Outcomes

With respect to the results of posttreatment gynecological examination (Fig. 1B), the cervix was visible and tumor invasion was not palpable, while the mucosa on the left side of the external orifice was gray and altered after treatment. Pelvic magnetic resonance imaging reexamination revealed that the original cervical signal shadow had largely disappeared (Fig. 2C, D).

## 3. Conclusion

In patients with a history of breast cancer, gynecological examination should be conducted when patients experience symptoms such as vaginal bleeding or abnormal vaginal leucorrhea. Cytological examination, colposcopy, and pathological examination should be performed, particularly when the cervical pathological report indicates the presence of an adenocarcinoma. IHC staining should be conducted to exclude the presence of secondary cervical metastases, and in cases when patients present with local cervical metastases that are inoperable, 3-dimensional brachytherapy holds promise as a relatively novel treatment approach. We hope that this report and the accompanying review help to enrich the literature pertaining to the treatment of rare cervical metastases, providing a foundation for the improved survival of affected patients.

## 4. Discussion

The cervical metastasis of tumors other than those of the female reproductive tract is very rare, with the most common primary tumors associated with such metastases being colon, endometrial, breast, appendix, and gastric tumors.^[5]^ Breast cancer most commonly metastasizes the lungs, liver, and bone, with metastases to the reproductive organs or cervix being rare, while ovarian metastasis is common. Pomerance and Mackles and Abell and Gosling,^[3,4]^ respectively, summarized findings from 223 and 237 cervical adenocarcinoma patients, reporting overall incidence rates of breast cancer metastasis to the cervix of 0.8% and 1.7%, respectively. In a 65-year review, Lemoine and Hall^[6]^ reported that 4 (12%) of 332 cases of cervical metastatic cancer were associated with primary breast tumors. Cochrane et al^[7]^ described 35 prior cases and 1 new case reported in the international literature, while Wang et al^[8]^ summarized 6 cases of such metastases in China. Most of these prior reports are case reports offering a detailed overview of the presentation of cervical breast cancer metastases. Based on a review of the extant literature, it appears that cervical metastases are likely rare at least in part because the cervix is a small organ that is rich in fibrous connective tissue with a relatively limited blood supply and only lymphatic drainage, making it less conducive to metastasis.

With respect to the clinical manifestations associated with these cases, the majority of patients experience abnormal vaginal bleeding, abnormal vaginal secretions, lower abdominal pain, and related symptoms. Yazigi et al^[9]^ reported a series of cases together with 24 cases that had previously been reported as of 1998. Kennebeck and Alagoz^[10]^ and Green et al^[11]^ reported 4 further cases, with vaginal bleeding affecting 17 of these 28 cases, while just 6 patients were asymptomatic. Hepp et al^[12]^ reported that vaginal bleeding was the most common clinical symptom among 27 patients with cervical metastases and primary breast cancer, impacting 57% of these patients, whereas 32% exhibited no clinical signs, and Pap smear results failed to detect any evidence of cervical malignancy in one-third of these cases. Wang et al^[8]^ summarized 6 cases in China, including 4 in which the patients experienced vaginal bleeding or abnormal vaginal secretion. In the present case, the patient did not exhibit any abnormal vaginal bleeding, although she did experience postmenopausal vaginal discharge. The patient’s vaginal thinprep cytologic test was normal. A review of the pathological typing results for cervical primary breast cancer metastases revealed that lobular carcinoma was the most common histologic type (50%), while ductal carcinoma accounted for 33.3% of cases, although based on their results from 10 cases of metastatic cervical cancer, Pérez-Montiel^[2]^ concluded that ductal carcinoma was the most common histological type. In line with this possibility, ductal carcinoma was the pathology finding after the initial procedure for the patient in the present case.

The clinical presentation of secondary metastatic cervical tumors is generally consistent with that of primary cervical tumors, with vaginal bleeding being a particularly common finding. Auxiliary examination revealed abnormal Pap smear results, imaging findings suggested a cervical mass, and gynecological examination results were consistent with the presence of cervical lesions. As the treatment approaches for primary and secondary cervical tumors differ markedly, it is important to effectively differentiate between the two. IHC results play a key role in this differentiation process. Cytokeratin 7 (CK7) is a type II keratin associated with nonkeratinizing epithelium. A CK7+/CK20 + IHC pattern is observed in some cervical cancer cases, whereas CK7+/CK20− staining can be associated with breast, cervical, and endometrial tumors.^[13]^ IHC results in the present case were CK7+/CK20−. When the primary tumor of origin is unknown, supplementary markers can aid in the identification of tumors of mammary origin including GATA3 (binding protein), mammaglobin (lactoglobulin), and GCDFP15 (giant cystic disease liquid protein). In the present case, IHC results included GATA3 (3 +) and mammaglobin (individual +) but GCDFP15 (−), thereby helping to clarify the final diagnosis.

Relatively little has been reported with respect to how best to treat cervical metastases associated with primary breast tumors. Green et al,^[11]^ Cruz,^[14]^ Way,^[15]^ Bryson et al,^[16]^ and Mousavi and Karimi Zarchi^[17]^ suggest that when the cervix is the only site of metastasis, good patient quality of life can be maintained following surgical resection of the metastatic lesion. Ma and Sun^[18]^ reported that radiotherapy alone should be used to treat patients with cervical breast cancer metastases, with the patient in their cases surviving for 20 months after treatment with an external irradiation dose of 40 to 45 Gy (mainly total pelvic irradiation) combined with an intracavitary irradiation dose of about 35 Gy at point A. The average duration of survival for patients with cervical metastases has been reported as 28 months (range: 2 months–12 years). In prior reports, isolated cervical metastases have been treated via surgical resection, but in the present case, the patient exhibited multiple metastases throughout her body such that complete surgical resection was regarded as infeasible, and these past cases thus provide no relevant reference. Following a multidisciplinary team discussion, the patient was administered local 3-dimensional brachytherapy, and after 5 rounds of treatment, the abnormal surgical signal had largely disappeared. Following 6 rounds of cavity treatment, the appearance of the cervix was visible to the naked eye with the tumor having largely disappeared such that the local treatment of the cervix achieved a complete response. This approach thus represents a promising new strategy for the management of patients with inoperable local cervical metastases. Following the completion of local radiotherapy of the cervix, the patient continued to undergo systematic treatment in the breast department and ultimately developed esophageal metastases over the course of follow-up treatment. This led to eating restrictions such that the patient died after 4 months of nutritional support and palliative treatment. Like this most of the case reports are descriptive studies, based on the treatment outcome of the clinical cases themselves, so the study design is not rigorous enough, there is no standardized control design, and the demonstration intensity of clinical hypothesis is low. The case report is a summative study of clinical experience, which can be used to propose scientific hypotheses, but it is usually impossible to verify the scientific hypotheses.

## Author contributions

**Writing—original draft:** Lu Zhang.

**Supervision:** Jusheng An, Manni Huang.

**Writing—review & editing:** Jusheng An, Manni Huang.

**Methodology:** Xi Yang.

**Resources:** Yuanyuan Zhang, Yaru Wen.
